# Tetanus and Hydrophobia

**Published:** 1896-11

**Authors:** W. A. Hillebrecht

**Affiliations:** Brooklyn, N. Y.


### TETANUS AND HYDROPHOBIA.

BROOKLYN, N. Y., October 3, 1896.

In a recent case of tetanus which I saw and which died, the thought occurred to me at the autopsy whether the cerebro-spinal fluid of this case inoculated into a rabbit would cause tetanus as does hydrophobia. The differential diagnosis between tetanus and hydrophobia being, no hallucinations present in the former, while in the latter they are marked symptoms. I would like the opinions of your readers on this subject. Kindly present same in your JOURNAL, and greatly oblige.

With regards, yours very truly,

W. A. HILLEBRECHT, B.S., M.D.

---

### THE TREATMENT OF SWELLED TESTICLE.

In the third volume of Dennis and Billings's new *System of Surgery*, Dr. J. William White and Dr. William H. Furness, 3d, the authors of the section on the "Surgery of the Genito-Urinary System," remark that a patient in whom epididymitis is developing should be put to bed at once, with the scrotum elevated on a pad of cotton. Free movements of the bowels should be kept up and the diet restricted to the very lightest food. If there is any fever, it is well to give a drop of tincture of aconite and five grains of potassium bromide every two or three hours. The affected testicle should be wrapped in lint and kept constantly moistened either with lead-water and laudanum, or with the following lotion:

Tincture of aconite,
Tincture of opium, each .................................................. 1 fl. oz.
Diluted lead water,
Water, each .................................................................. 2 fl. oz.

Cold compresses (not iced) are often very soothing, but rest is the chief curative measure during the acute stage.—*New York Med. Jour.*

